# Casticin Alleviates Acetaminophen-Induced Acute Liver Injury by Modulating the TLR4/MyD88/TRAF6/NF-κB Signaling Pathway

**DOI:** 10.3390/ph19071111

**Published:** 2026-07-18

**Authors:** Salman H. Alotaibi, Mahmoud M. Samaha, Manar G. Helal, Dina S. El-Agamy

**Affiliations:** Department of Pharmacology and Toxicology, Faculty of Pharmacy, Mansoura University, Mansoura 35516, Egypt; xr.2@hotmail.com (S.H.A.); manargamal@mans.edu.eg (M.G.H.)

**Keywords:** drug-induced liver injury, oxidative stress, inflammation, hepatoprotection, flavonoids

## Abstract

**Background:** Acute liver injury (ALI) is commonly caused by acetaminophen (APAP) overdose, which drives oxidative stress alongside activation of innate immune signaling. Casticin, a naturally occurring flavonoid, has anti-inflammatory and antioxidant properties. Focusing on the toll-like receptor 4 (TLR4)/myeloid differentiation primary response 88 (MyD88)/tumor necrosis factor receptor-associated factor 6 (TRAF6)/nuclear factor kappa B (NF-κB) pathway, this study assessed casticin’s ability to protect mice from APAP-induced hepatotoxicity. **Methods:** APAP-induced ALI was established in mice randomly assigned to the following six groups: normal control, casticin control, APAP, APAP plus N-acetylcysteine (NAC), APAP plus low-dose casticin, and APAP plus high-dose casticin. Casticin was administered for three consecutive days before APAP to evaluate its preventive rather than therapeutic potential. Biochemical and histological analyses were performed, with molecular assessments using Western blotting, ELISA, quantitative real-time PCR (qPCR), and immunohistochemistry. **Results:** APAP significantly elevated serum ALT, AST, and ALP and markedly deteriorated hepatic architecture, confirming hepatotoxicity. APAP also induced lipid peroxidation and depleted antioxidant defenses. Hepatic TNF-α and IL-6 increased, IL-10 decreased, and the abundance of TLR4, MyD88, TRAF6, and NF-κB p65 was elevated. Casticin reduced these pathway components, lowered TNF-α and IL-6, and increased IL-10 dose-dependently, with effects approaching those of NAC. **Conclusions:** Casticin protected the liver against APAP toxicity, restraining oxidative injury while damping TLR4/MyD88/TRAF6/NF-κB signaling.

## 1. Introduction

Among the causes of acute liver failure (ALF), drug-induced liver injury (DILI) ranks high and continues to impose a substantial burden worldwide. Acute liver injury (ALI), the form in which DILI most often presents, involves rapid destruction of hepatocytes together with pronounced oxidative stress and a vigorous inflammatory response. Recovery is common, yet severe or progressive disease may descend into ALF, in which hepatic function is profoundly lost and life is threatened. Supportive care has advanced; pharmacological means of preventing or reversing ALI have not, and the search for new hepatoprotective agents remains open [1].

APAP, known also as paracetamol, ranks among the most frequently consumed antipyretic and analgesic medicines worldwide. Its therapeutic window is wide, but ingestion of supratherapeutic amounts accounts for the largest share of drug-induced ALI in a number of countries. Experimental APAP overdose has consequently become a standard preclinical platform on which the pathogenesis of hepatic injury is dissected, and candidate hepatoprotective molecules are screened [2].

Hepatocellular toxicity originates in how the drug is handled metabolically within the liver. At therapeutic exposures the bulk of an APAP dose leaves the hepatocyte as innocuous glucuronide and sulfate conjugates. A minor fraction is instead oxidized by cytochrome P450 isoenzymes—CYP2E1 above all—yielding N-acetyl-p-benzoquinone imine (NAPQI). This species is short-lived and strongly electrophilic, and in ordinary circumstances reduced glutathione (GSH) quenches it by conjugation before harm is done. However, during APAP overdose or under conditions in which oxidative metabolism predominates and/or hepatic GSH reserves are depleted, NAPQI accumulates, resulting in excessive reactive oxygen species (ROS) generation, mitochondrial dysfunction, and covalent binding to cellular proteins. Excessive NAPQI also binds to mitochondrial proteins, leading to mitochondrial oxidative stress, sustained activation of c-Jun N-terminal kinase (JNK), hepatocellular necrosis, and acute liver injury (ALI) [3,4,5,6].

Once hepatocytes are injured, damage-associated molecular patterns (DAMPs) are released into the extracellular space and set off a sterile inflammatory response. Toll-like receptor 4 (TLR4) is among the innate immune sensors implicated in that response as follows: its engagement draws in myeloid differentiation primary response 88 (MyD88), activates tumor necrosis factor receptor-associated factor 6 (TRAF6), and ultimately permits nuclear factor kappa B (NF-κB) to transcribe pro-inflammatory cytokine genes, among them tumor necrosis factor-alpha (TNF-α) and interleukin-6 (IL-6). Whether this signaling is itself a driver of APAP hepatotoxicity, or instead a downstream consequence of necrosis, is still argued. Either way, tempering the pathway may slow the progression of injury [7,8,9,10].

Plant-derived flavonoids have drawn attention as candidate protectants in drug-induced liver injury, acting at once on redox balance, mitochondrial integrity, and inflammatory signaling. Quercetin, luteolin, baicalein, naringenin, and isorhamnetin each blunt hepatotoxicity by curbing reactive oxygen species production, safeguarding mitochondria, bolstering endogenous antioxidant defenses, and dampening TLR4/MyD88/TRAF6/NF-κB-driven inflammation. The two arms are not independent: mitochondrial oxidant stress feeds sterile inflammatory signaling, and the pair together accelerate hepatic injury [10,11,12,13].

One such compound is casticin, a polymethoxylated flavonoid isolated from species of the genus Vitex. Its documented pharmacological repertoire spans cytoprotective, antioxidant, and anti-inflammatory activity. Earlier work indicates that casticin engages several intracellular cascades governing redox balance and inflammatory tone—a profile consistent with protection of tissue placed under toxic stress. Despite these advances, mechanistic evidence regarding casticin in acetaminophen-induced acute liver injury remains limited. To the best of our knowledge, no previous study has comprehensively evaluated the hepatoprotective effect of casticin in relation to oxidative stress together with the TLR4/MyD88/TRAF6/NF-κB inflammatory pathway in an APAP-induced liver injury model. Therefore, the present study aimed to investigate whether casticin attenuates APAP-induced ALI through coordinated modulation of oxidative stress and inflammation associated with this pathway [14,15,16].

## 2. Results

Administration of casticin alone (20 mg/kg) did not produce any significant changes in the evaluated biochemical, oxidative stress, inflammatory, molecular, or histopathological parameters compared with the normal control group, indicating that the selected dose was well-tolerated and showed no evidence of treatment-related toxicity.

### 2.1. Impact of Casticin on APAP-Induced Hepatic Histopathological Lesions

**Figure 1:**
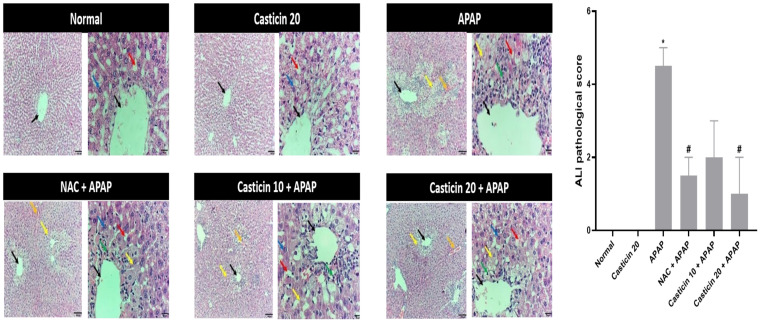
Impact of casticin on hepatic histopathology after APAP challenge. Representative H and E-stained liver sections and the corresponding ALI pathological scores. Arrows: black, central vein; red, sinusoids containing Kupffer cells; blue, polyhedral hepatocytes; green, inflammatory cells; yellow, centrilobular necrosis; orange, portal vessel congestion. Magnification 100× (scale bar 100 µm) and 400× (scale bar 20 µm). Scores were presented as median and statistically analyzed using *Kruskal–Wallis* non-parametric test followed by *Dunn’s* multiple comparison test (*n* = 8). * *p* < 0.05 versus the normal control group and ^#^ *p* < 0.05 versus the APAP group, respectively.

As shown in Figure 1, the six groups differed markedly on histological examination. Parenchyma from the normal and casticin 20 animals was intact and scored 0. APAP produced severe damage, scored 4, with widespread inflammatory infiltration, vascular congestion, hepatocellular vacuolation, and centrilobular necrosis. NAC pretreatment tempered these changes considerably and brought the injury score down to 2. Casticin likewise improved hepatic morphology and did so dose-dependently: the low dose left moderate injury (score 2), whereas the high dose permitted only minimal alteration, scoring 1.

### 2.2. Impact of Casticin on Liver Function Biomarkers

Serum ALT, AST, and ALP activities rose sharply in APAP-challenged mice relative to normal controls. Pretreatment with NAC or either dose of casticin significantly reduced all three enzyme activities relative to the APAP group (Table 1).

### 2.3. Impact of Casticin on Hepatic Oxidant/Antioxidant Balance

As presented in Table 2, hepatic malondialdehyde (MDA) accumulated markedly after APAP relative to normal controls. This accumulation was significantly blunted by NAC and by both casticin doses. Hepatic GSH followed the opposite trajectory as follows: stores were severely depleted in the APAP group, whereas NAC and either casticin dose restored tissue GSH toward control values.

### 2.4. Impact of Casticin on Hepatic TLR4 Expression

**Figure 2:**
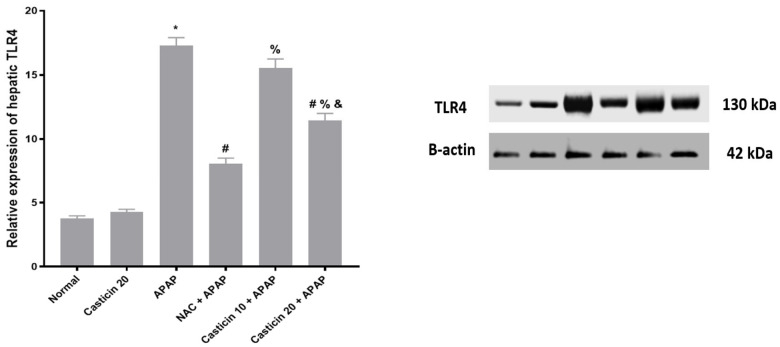
Impact of casticin on hepatic TLR4 expression. Values are means ± SE (*n* = 3); * versus normal, ^#^ versus APAP, ^%^ versus NAC + APAP, and ^&^ versus casticin 10 + APAP, respectively using one-way ANOVA followed by Tukey–Kramer multiple comparisons post hoc test.

Hepatic TLR4 protein abundance was clearly higher in APAP-treated mice than in normal animals. Pretreatment with NAC or with 20 mg/kg casticin lowered TLR4 significantly relative to the APAP group, whereas the 10 mg/kg dose produced a downward shift that did not reach statistical significance (Figure 2).

### 2.5. Impact of Casticin on Hepatic MyD88 Expression

**Figure 3:**
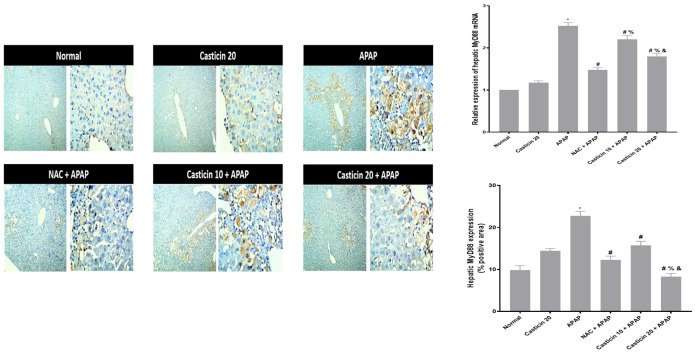
Impact of casticin on hepatic MyD88 expression. Values are means ± SE (*n* = 8); * versus normal, ^#^ versus APAP, ^%^ versus NAC + APAP, and ^&^ versus casticin 10 + APAP, respectively using one-way ANOVA followed by Tukey–Kramer multiple comparisons post hoc test.

Hepatic MyD88 rose significantly in APAP-treated mice relative to normal controls, a finding returned independently by qPCR and by immunohistochemistry. NAC and both casticin doses lowered MyD88 relative to APAP, although the two methods differed slightly in the magnitude of reduction they registered, most noticeably in the low-dose casticin group (Figure 3).

### 2.6. Impact of Casticin on Hepatic TRAF6 Expression

**Figure 4:**
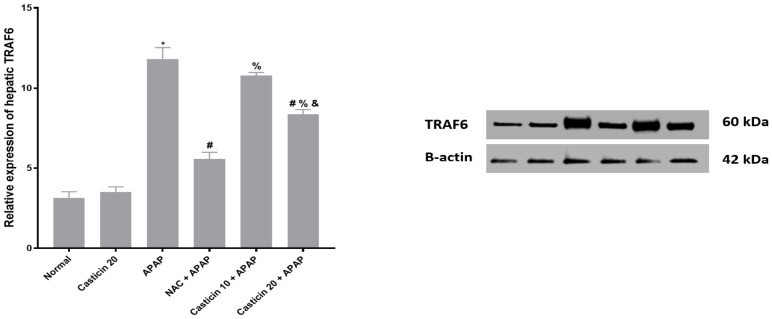
Impact of casticin on hepatic TRAF6 expression. Values are means ± SE (*n* = 3); * versus normal, ^#^ versus APAP, ^%^ versus NAC + APAP, and ^&^ versus casticin 10 + APAP, respectively using one-way ANOVA followed by Tukey–Kramer multiple comparisons post hoc test.

TRAF6 protein in liver tissue increased appreciably following APAP administration. A significant reduction was recorded in the NAC and 20 mg/kg casticin groups, while the decrease seen with 10 mg/kg casticin fell short of significance when compared with APAP alone (Figure 4).

### 2.7. Impact of Casticin on Hepatic NF-κB p65 Expression

**Figure 5:**
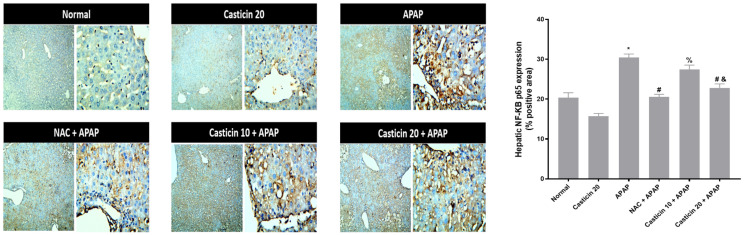
Impact of casticin on hepatic NF-κB p65 expression. Values are means ± SE (*n* = 8); * versus normal, ^#^ versus APAP, ^%^ versus NAC + APAP, and ^&^ versus casticin 10 + APAP, respectively using one-way ANOVA followed by Tukey–Kramer multiple comparisons post hoc test.

As presented in Figure 5, hepatic NF-κB p65 was markedly elevated in APAP-exposed mice compared with normal controls. Pretreatment with NAC or with high-dose casticin lowered p65 significantly relative to APAP, whereas the reduction achieved by the low dose was not statistically significant.

### 2.8. Impact of Casticin on Hepatic Pro-Inflammatory/Anti-Inflammatory Biomarkers Expressions

**Figure 6:**
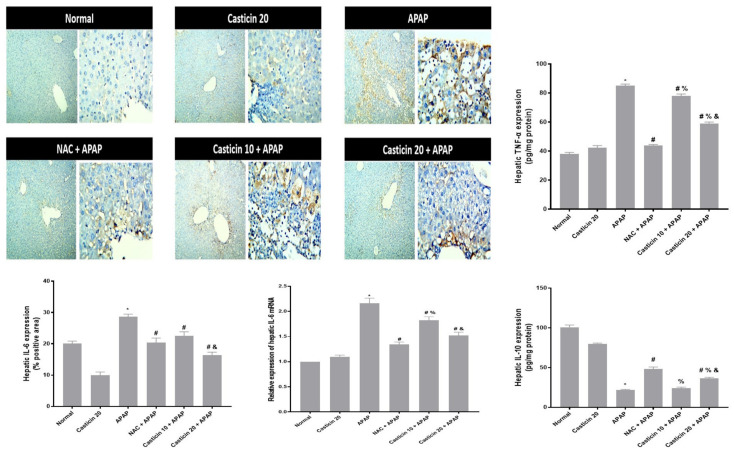
Impact of casticin on hepatic pro-inflammatory/anti-inflammatory biomarkers expressions. Values are means ± SE (*n* = 8); * versus normal, ^#^ versus APAP, ^%^ versus NAC + APAP, and ^&^ versus casticin 10 + APAP, respectively using one-way ANOVA followed by Tukey–Kramer multiple comparisons post hoc test.

APAP raised hepatic TNF-α and IL-6 well above control values, and NAC as well as either casticin dose brought each cytokine back down significantly. IL-10 behaved inversely, collapsing after APAP. NAC and casticin 20 mg/kg restored it significantly, whereas 10 mg/kg produced only a modest, non-significant rise (Figure 6).

## 3. Discussion

The present data show casticin acting on two fronts at once against APAP hepatotoxicity, one antioxidant and one anti-inflammatory. Histopathology, serum biochemistry, and molecular readouts converged on the same conclusion as follows: hepatic damage was substantially reduced, most clearly at the higher dose, which is what a multi-target agent would be expected to produce.

In the current study, APAP administration resulted in severe hepatic injury characterized by inflammatory cell infiltration, portal congestion, vacuolation, and centrilobular necrosis, which are hallmark features of APAP-induced hepatotoxicity as previously documented [17,18]. NAC treatment, a standard treatment for APAP-induced injury, produced partial improvement, with mild residual structural alterations. 

Casticin demonstrated a dose-related protective effect, where the low dose showed moderate improvement, while the high dose markedly preserved hepatic architecture with minimal pathological changes. These structural improvements likely reflect the ability of casticin to limit both hepatocellular degeneration and inflammatory infiltration, thereby maintaining tissue integrity under toxic insult. The histological picture was mirrored biochemically: serum ALT, AST, and ALP all climbed after APAP, consistent with hepatocellular membrane damage and loss of functional capacity, and in keeping with earlier reports [18,19,20]. Pretreatment with casticin tempered each of these enzyme rises. Read alongside the preserved lobular architecture on histology, the enzyme data suggest that casticin sustains hepatocyte functional integrity rather than merely limiting overt structural collapse. Comparable protection has been described for casticin in other models of chemical-induced liver injuries, including cisplatin- and methotrexate-associated hepatotoxicity and carbon tetrachloride-driven fibrosis [21,22,23]. Having established a protective signal in the APAP model, we next examined the mechanisms that might underlie it.

Redox imbalance sits close to the center of APAP hepatotoxicity and accounts for much of the ensuing necrosis and functional failure. Once CYP2E1-derived NAPQI exhausts the hepatic GSH pool, the unquenched metabolite adducts mitochondrial proteins and provokes a surge of ROS [24,25]. What follows is a cascade: membrane lipids peroxidize, the mitochondrial permeability transition pore opens, ATP synthesis fails, DNA fragments, stress-activated kinases are engaged, and the hepatocyte dies [26]. Mitochondrial oxidant stress additionally amplifies the inflammatory limb of the injury, since DAMP release and redox-sensitive transcription factors such as NF-κB are both engaged under these conditions [27,28].

In the current study, APAP markedly increased hepatic MDA levels and reduced GSH content, confirming enhanced lipid peroxidation and impaired antioxidant defense. Importantly, pretreatment with casticin attenuated MDA elevation and restored GSH levels, indicating improved redox balance. This is particularly relevant, as lipid peroxidation compromises hepatocyte membrane integrity, leading to cell death. By preserving GSH levels, casticin enhances cellular resistance to the reactive metabolite NAPQI, thereby limiting early oxidative damage. These observations sit alongside a body of earlier work describing antioxidant activity for casticin across unrelated disease contexts, spanning allergic airway inflammation, cisplatin nephrotoxicity, osteoarthritis, and degenerative osteoarticular disease [29,30,31,32].

A second amplifying loop is immunological, and the TLR4/MyD88/TRAF6/NF-κB axis occupies a central position within it [33]. Hepatocytes killed by NAPQI-driven oxidant and mitochondrial injury spill DAMPs—HMGB1 and heat-shock proteins prominent among them—into the sinusoidal space, where they ligate TLR4 displayed by Kupffer cells and recruited leukocytes. Engaged TLR4 recruits the adaptor MyD88; TRAF6 and the IκB kinase complex are activated in turn, and NF-κB, released from inhibition, enters the nucleus, where it switches on pro-inflammatory genes, TNF-α and IL-6 included [34,35]. Where this loop runs unchecked, leukocyte influx, oxidant burden, and necrosis reinforce one another and hepatic function deteriorates further. Our data are consistent with activation of precisely this cascade, since APAP raised both transcript and protein levels of the pathway components together with their downstream cytokines. Casticin blunted the cascade at several points as follows: the 20 mg/kg dose lowered TLR4, TRAF6, and NF-κB p65, and both doses reduced MyD88, implying interference with initiation as well as with propagation of the signal. The cytokine profile shifted accordingly, with TNF-α and IL-6 falling and IL-10 rising at the higher dose—a movement toward resolution rather than perpetuation of inflammation, which plausibly limits secondary tissue damage [15]. Suppression of NF-κB signaling by casticin has likewise been reported in unrelated models of inflammatory disease [30,32,36,37].

When compared with NAC, casticin exhibited comparable protective effects on hepatic architecture and inflammatory signaling. Although NAC showed a greater tendency to normalize certain liver function and oxidative stress markers, high-dose casticin produced comparable improvements across several evaluated endpoints, particularly in preserving hepatic histological architecture. Considering the well-established but time-dependent efficacy of NAC in APAP intoxication, casticin represents a promising experimental candidate with combined antioxidant and anti-inflammatory properties.

## 4. Materials and Methods

### 4.1. Animals

Forty-eight adult male Swiss Albino mice (25 ± 5 g) were supplied by the Urology and Nephrology Center, Mansoura University. Animals were acclimatized in the institutional animal facility for two weeks before use and were maintained under standard housing conditions throughout the experiment. All procedures were approved by the Faculty of Pharmacy Research Ethics Committee under the oversight of the Mansoura University Animal Care and Use Committee, Egypt (approval number: MU-ACUC (PHARM.PhD.24.01.34)). Handling of animals followed the NIH Guide for the Care and Use of Laboratory Animals (Publication No. 85-23, revised 1985), and reporting conforms to the ARRIVE 2.0 guidelines.

### 4.2. Drugs and Chemicals

Casticin was bought as powder (#479-91-4, Cayman Chemical Co., Ann Arbor, MI, USA, purity of ≥98%), while N-acetylcysteine (NAC) was supplied as Acetylcistein^®^ effervescent granules (Sedico, 6th of October City, Egypt), and APAP as Injectmol^®^ solution (Pharma-Tech, Cairo, Egypt). Casticin and NAC were each suspended in 0.5% (*w*/*v*) sodium carboxymethylcellulose (CMC-Na) for oral dosing. All the other chemicals used in the study were of high grade and analytical purity.

### 4.3. Experimental Protocol

ALI was induced by single intraperitoneal (i.p.) injection of APAP (400 mg/kg) as previously reported [19,38]. Mice were allocated to the experimental groups using a simple randomization method (6 groups/8 mice each) and treated as follows: Normal: mice received 0.5% CMC-Na orally followed by an intraperitoneal injection of normal saline; Casticin 20: mice received only high dose of casticin (20 mg/kg); APAP: mice received APAP; NAC + APAP: mice received N-acetyl cysteine (NAC, 400 mg/kg) [39] followed by APAP; Casticin 10 + APAP: mice received a low dose of casticin (10 mg/kg) followed by APAP; and Casticin 20 + APAP: mice received of high dose of casticin (20 mg/kg, orally) followed by APAP. The selected doses of casticin were based on previous pharmacological studies demonstrating significant anti-inflammatory and antioxidant effects without evidence of systemic toxicity [40,41].

Mice were given casticin (10 or 20 mg/kg), NAC or an equivalent amount of CMC-Na by oral gavage (0.25 mL/mouse) for 3 consecutive days at the same time, and after 3 h of the last dose, a single i.p. injection of APAP (400 mg/kg; 1 mL/mouse) was administered. Twenty-four hours after APAP injection, mice were anesthetized with thiopental sodium (40 mg/kg, i.p.). Blood samples were collected from the retro-orbital venous plexus and allowed to clot before centrifugation at 4000 rpm for 15 min. The separated serum was stored at −80 °C until biochemical analysis. Following blood collection, mice were euthanized by exsanguination under deep thiopental anesthesia, and the liver was immediately excised and separated into lobes. Left hepatic lobes were immersion-fixed in 10% (*v*/*v*) neutral-buffered formalin and reserved for histopathology and immunohistochemistry. From the right lobe, a weighed portion was homogenized in phosphate-buffered saline (10% *w*/*v*) and clarified by centrifugation (4000 rpm, 15 min, 4 °C); the supernatant was aliquoted and held at −80 °C for biochemical assays and enzyme-linked immunosorbent assays (ELISAs). A further portion of the right median lobe was snap-frozen in liquid nitrogen and stored at −80 °C pending qPCR and Western blot techniques analysis. All biochemical, molecular, immunohistochemical, and histopathological analyses were performed by investigators blinded to the group allocation.

### 4.4. Liver Function Biomarkers

Serum ALT, AST, and ALP activities were quantified colorimetrically using commercial kits (Biodiagnostic, Giza, Egypt) according to the supplier’s instructions.

### 4.5. Hepatic Oxidant/Antioxidant Parameters

Hepatic GSH and MDA were measured in tissue homogenates with commercial colorimetric kits from the same supplier (Biodiagnostic, Giza, Egypt), following the accompanying protocols. The results are expressed as mg/g tissue for GSH and nmol/g tissue for MDA.

### 4.6. Hepatic TNF-α and IL-10 Expressions

According to the manufacturer’s instructions, hepatic levels of TNF-α (#CSB-E04741m) and IL-10 (#CSB-E04594m-IS) were assessed using commercially available ELISA kits (Cusabio, Houston, TX, USA).

### 4.7. Western Blot Analysis for Hepatic TLR4 and TRAF6 Expressions

Protein was recovered from liver tissue with TriFast reagent (Peqlab, VWR), a single reagent from which RNA, DNA, and protein can each be isolated. Tissue (50–100 mg) was homogenized in 1 mL TriFast, chloroform was added at 0.2 mL per 1 mL TriFast, and the mixture was centrifuged (12,000× *g*, 5 min). The aqueous layer was discarded and the interphase and organic phases carried forward for protein recovery. Isopropanol precipitation followed (12,000× *g*, 10 min, 4 °C). The pellet was washed three times with 0.3 M guanidine hydrochloride in 95% ethanol and then with 100% ethanol (7500× *g*, 5 min, 4 °C for each wash), dried under vacuum, and taken up in 1% SDS. Protein content was assayed by the Bradford method, reading absorbance at 595 nm after reaction with Coomassie Brilliant Blue G-250 and quantifying against bovine serum albumin standards.

Samples were resolved at 30 µg of protein each on a hand-cast SDS-polyacrylamide gel (10% resolving, 4% stacking) alongside a PageRuler Unstained Protein Ladder. A potential of 75 V carried the samples through the stacking layer, after which 125 V was applied for roughly 2 h. Proteins were then moved to a Hybond nylon membrane (GE Healthcare) in a wet tank at 100 V over 1.5 h. Non-specific binding was suppressed by a 1 h incubation at room temperature in 5% non-fat dry milk made up in Tris-buffered saline containing 0.1% Tween-20. Membranes then sat overnight at 4 °C with primary antibodies raised against TLR4 (Abcam, Cambridge, UK, #ab217274, 1:1000, 130 kDa), TRAF6 (Abcam, UK, #ab137452, 1:2000, 60 kDa), or β-actin (Abcam, UK, #ab8227, 1:3500, 42 kDa). After washing, an HRP-conjugated secondary antibody (Novus Biologicals, Centennial, CO, USA, 1:7500) was applied for 1 h at room temperature and the membranes washed once more. Signal was captured on a Geldoc-it imaging system (UVP, Upland, CA, USA) under automatic exposure, band intensities were measured in TotalLab software (v1.0.1), and target protein levels were expressed relative to β-actin as loading control. All Western blot analyses were conducted on three independent biological replicates per experimental group to ensure reproducibility.

### 4.8. Quantitative Real-Time PCR (qPCR)

Hepatic total RNA was isolated with the RNeasy Mini Kit (Qiagen, Hilden, Germany). One microgram of RNA per sample was reverse-transcribed with the RevertAid First Strand cDNA Synthesis Kit (Thermo Scientific, Rockford, IL, USA) as directed by the supplier. Amplification was carried out with the HERA SYBR Green qPCR Kit (Willowfort, Birmingham, England) on a real-time thermal cycler (Thermo Fisher Scientific, Vantaa, Finland). Relative expression of myeloid differentiation primary response 88 (MyD88) and interleukin-6 (IL-6) mRNA were calculated by the 2^−ΔΔCt^ method using glyceraldehyde 3-phosphate dehydrogenase (GAPDH) mRNA expression as the internal standard. The primers used were as follows: MyD88 forward, 5′-GAGATCCGCGAGTTTGAGAC-3′ and reverse, 5′-TTGTCTGTGGGACACTGCTC-3′ and IL-6 forward, 5′-TCCTACCCCAACTTCCAATGCTC-3′ and reverse, 5′-TTGGATGGTCTTGGTCCTTAGCC-3′ and GAPDH forward, 5′-AACGGATTTGGTCGTATTGGG-3′ and reverse, 5′-TCGCTCCTGGAAGATGGTGAT-3′.

### 4.9. Histopathological and Immunohistochemical Examination

Fixed liver tissue was prepared for histopathological evaluation of hematoxylin and eosin (H and E) stained samples and immunohistochemistry investigation. Histopathological examination of H and E-stained sections for liver injury was conducted blindly and randomly by a histopathologist, utilizing the grading system adopted by **Muhammad-Azam et al.** [42]. On this scale, increasing scores denote progressively wider necrotic involvement of the hepatic lobule, from normal parenchyma (0), through focal centrilobular necrosis and expanding midzonal and centrilobular-portal involvement, to extensive hepatocyte loss (5). Images were captured under 10× and 40× objectives on a MEIJI MX5200L microscope (Saitama, Japan) fitted with an MVV5000CL digital eyepiece, using Future WinJoe software version 1.6.5.

The expression levels of MyD88, NF-κB p65, and IL-6 in hepatic tissues were determined by immunostaining utilizing the Avidin-Biotin Complex method [43]. The following antibodies were recruited: MyD88 (Abcam, UK, #ab28763), NF-κB p65 (Invitrogen, Waltham, MA, USA, #PA5-16545), and IL-6 (Abcam, UK, #ab6672). IHC-positive cells were quantified in ImageJ from high-resolution images of the entire tissue section. Regions of interest were extracted from whole sections at magnifications of up to 40× so that relative antigen content could be assessed. Color deconvolution was then applied to separate the brown 3,3′-diaminobenzidine (DAB) chromogen from the hematoxylin counterstain. The resulting monochromatic DAB image was subjected to frequency analysis in ImageJ version 1.54 (FIJI, National Institutes of Health, Bethesda, MD, USA), where five random fields per section were captured, from which the DAB (antigen) area fraction was derived [44].

### 4.10. Statistical Analysis

Results are reported as mean ± standard error of the mean (SE), with *n* denoting the number of mice per group. All analyses, graphing, and fitting of ELISA standard curves were carried out in GraphPad Prism (GraphPad Software Inc. V8, San Diego, CA, USA). The *Shapiro–Wilk* test checked whether the data were normally distributed. A threshold of *p* < 0.05 defined significance throughout. Group differences were evaluated by one-way analysis of variance (ANOVA), with *Tukey–Kramer’s* multiple comparisons applied post hoc. Histopathological liver injury scores were reported as median and analyzed using the *Kruskal–Wallis* non-parametric test, followed by *Dunn’s* multiple comparison test.

## 5. Conclusions

**Figure 7:**
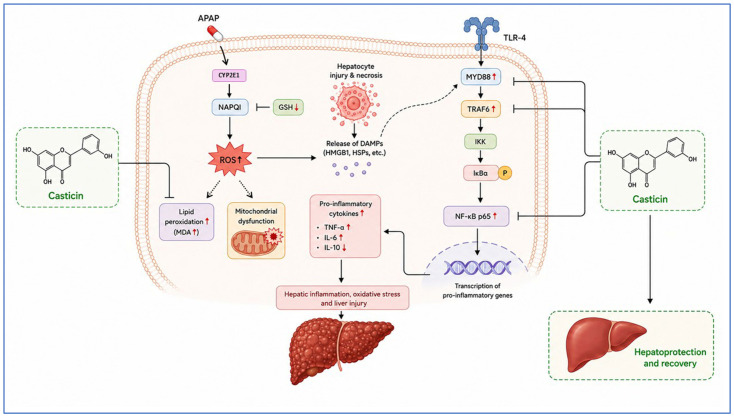
Proposed mechanisms of casticin-mediated hepatoprotection against acetaminophen-induced liver injury. The schematic illustration was generated using ChatGPT (GPT-5.6, OpenAI, San Francisco, CA, USA) based on the study findings and subsequently reviewed and edited by the authors for scientific accuracy.

Taken together, the data presented here establish casticin as an effective hepatoprotective agent in the APAP overdose model, with protection operating across two complementary axes (Figure 7). Redox first: casticin spared hepatic GSH reserves and held MDA accumulation in check, indicating attenuation of oxidative stress associated with APAP-induced liver injury. Turning to inflammation, casticin suppressed TLR4 and TRAF6 protein, brought down MyD88 and NF-κB p65, and lowered TNF-α and IL-6, while the anti-inflammatory cytokine IL-10 rose at the higher dose. This dual profile translated into measurable histopathological benefit, with high-dose casticin limiting centrilobular necrosis and inflammatory infiltration to a degree comparable with NAC, even though NAC produced a more complete normalization of serum enzyme activities. The differential response between casticin and NAC across outcome measures may reflect their distinct mechanisms as follows: NAC acts primarily by replenishing GSH, whereas casticin appears to simultaneously restrain innate immune signaling upstream of cytokine transcription. These results suggest that modulation of the TLR4/MyD88/TRAF6/NF-κB signaling pathway may contribute to the hepatoprotective effects of casticin.

## 6. Study Limitations

This work carries limitations that bear on how its findings should be read. Group sizes were modest, and casticin was assessed in only one murine APAP model; thus, how far the results generalize is uncertain. The design was preventive rather than curative, since dosing preceded the APAP challenge, which leaves efficacy after exposure untested. A short dosing window likewise says nothing about long-term efficacy or safety. Pharmacokinetics were not examined either. Casticin shares the liabilities of many plant flavonoids as follows: poor aqueous solubility, restricted intestinal uptake, heavy first-pass metabolism, and low systemic bioavailability, any of which could blunt therapeutic performance and complicate translation of the doses used here to clinical practice. Formulation may matter a great deal. Future work should therefore characterize the pharmacokinetic profile of casticin, explore delivery strategies such as nanoformulation to raise bioavailability and systemic exposure, and test efficacy in post-exposure models that mirror the clinical presentation of APAP overdose.

## Figures and Tables

**Table 1:** Impact of casticin on liver function biomarkers. Values are means ± SE (*n* = 8); * versus normal, ^#^ versus APAP, ^%^ versus NAC + APAP, and ^&^ versus casticin 10 + APAP, respectively using one-way ANOVA followed by Tukey–Kramer multiple comparisons post hoc test.

Experimental Group	ALT (IU/L)	AST (IU/L)	ALP (IU/L)
Normal	65.4 ± 4.1	181.8 ± 7.2	131.2 ± 3.7
Casticin 20	79.0 ± 2.2	218.8 ± 2.1	160.8 ± 2.3
APAP	3083.0 ± 141.8 *	3806 ± 85.1 *	1019 ± 18.5 *
NAC + APAP	104.4 ± 1.7 ^#^	217.4 ± 5.6 ^#^	179.6 ± 3.2 ^#^
Casticin 10 + APAP	946.6 ± 21.3 ^# %^	1169 ± 29.2 ^# %^	814.2 ± 6.4 ^# %^
Casticin 20 + APAP	258.4 ± 10.9 ^# &^	467.4 ± 11.3 ^# % &^	455.2 ± 8.1 ^# % &^

**Table 2:** Impact of casticin on hepatic oxidant/antioxidant balance. Values are means ± SE (*n* = 8); * versus normal, ^#^ versus APAP, ^%^ versus NAC + APAP, and ^&^ versus casticin 10 + APAP, respectively using one-way ANOVA followed by Tukey–Kramer multiple comparisons post hoc test.

Experimental Group	MDA (nmol/g Tissue)	GSH (mg/g Tissue)
Normal	16.7 ± 0.1	15.8 ± 0.3
Casticin 20	13.1 ± 0.2	17.7 ± 0.3
APAP	90.2 ± 3.3 *	2.6 ± 0.3 *
NAC + APAP	22.4 ± 1.6 ^#^	16.8 ± 0.5 ^#^
Casticin 10 + APAP	45.2 ± 1.6 ^# %^	5.3 ± 0.3 ^# %^
Casticin 20 + APAP	31.2 ± 0.4 ^# % &^	11.0 ± 0.2 ^# % &^

## Data Availability

All data generated or analyzed during this study are included in this published article.

## Abbreviations

The following abbreviations are used in this manuscript:

ALI	Acute liver injury
ALT	Alanine aminotransferase
APAP	Acetaminophen
AST	Aspartate aminotransferase
DAB	3,3′-diaminobenzidine
DAMPs	Damage-associated molecular patterns
DILI	Drug-induced liver injury
GAPDH	Glyceraldehyde-3-phosphate dehydrogenase
GSH	Reduced glutathione
H and E	Hematoxylin and eosin
i.p.	Intraperitoneal
IL	Interleukin
MDA	Malondialdehyde
MyD88	Myeloid differentiation primary response 88
NAC	N-acetylcysteine
NAPQI	N-Acetyl-p-benzoquinone imine
NF-κB	Nuclear factor kappa-B
ROS	Reactive oxygen species
TBST	Tris-buffered saline with Tween 20
TLR	Toll-like receptor
TNF	Tumor necrosis factor
TRAF	Tumor necrosis factor receptor-associated factor
